# Longitudinal population-based studies of affective disorders: Where to from here?

**DOI:** 10.1186/1471-244X-8-83

**Published:** 2008-09-23

**Authors:** John R Beard, Sandro Galea, David Vlahov

**Affiliations:** 1 New York Academy of Medicine, New York, New York, USA; 2 School of Public Health, University of Sydney, Sydney, Australia; 3 Faculty of Health and Applied Sciences, Southern Cross University, Lismore, Australia; 4 Department of Epidemiology, University of Michigan School of Public Health, Ann Arbor, MI, USA; 5 Department of Epidemiology, Columbia University Mailman School of Public Health, New York, NY, USA; 6 Department of Epidemiology, Johns Hopkins Bloomberg School of Public Health, Baltimore, MD, USA

## Abstract

**Background:**

Longitudinal, population-based, research is important if we are to better characterize the lifetime patterns and determinants of affective disorders. While studies of this type are becoming increasingly prevalent, there has been little discussion about the limitations of the methods commonly used.

**Methods:**

Discussion paper including a brief review of key prospective population-based studies as the basis for a critical appraisal of current approaches.

**Results:**

We identified a number of common methodological weaknesses that restrict the potential of longitudinal research to characterize the diversity, prognosis, and determinants of affective disorders over time. Most studies using comprehensive diagnostic instruments have either been of relatively brief duration, or have suffered from long periods between waves. Most etiologic research has focused on first onset diagnoses, although these may be relatively uncommon after early adulthood and the burden of mental disorders falls more heavily on individuals with recurring disorders. Analysis has tended to be based on changes in diagnostic status rather than anges in symptom levels, limiting study power. Diagnoses have generally been treated as homogeneous entities and few studies have explored whether diagnostic subtypes such as atypical depression vary in their etiology or prognosis. Little research has considered whether there are distinct trajectories of symptoms over time and most has focused on individual disorders such as depression, rather than considering the relationship over time between symptoms of different affective disorders. There has also been limited longitudinal research on factors in the physical or social environment that may influence the onset, recurrence or chronicity of symptoms.

**Conclusion:**

Many important, and in some respects quite basic, questions remain about the trajectory of depression and anxiety disorders over the life course and the factors that influence their incidence, recurrence and prognosis. Innovative approaches that consider symptoms of all affective disorders, and how these change over time, has the potential to greatly increase our understanding of the heterogeneity of these important conditions and of the individual and environmental characteristics that influence their life course.

Using longitudinal research to define sub classes of affective disorders may also be of great benefit for studies seeking to define the genetic determinants of susceptibility to these conditions.

## Background

Mental disorders are among the conditions causing the greatest burden on human health, with estimates in developed countries of the 12 month prevalence of depression alone ranging between 3.1% and 10.3% percent of the general population. [1,2] However, despite the demonstrated effectiveness of a range of interventions, many individuals with mental disorders will never seek clinical help[3,4]. This highlights the need for population based epidemiologic research, since study populations drawn only from clinical settings may not be representative of the broader community.

Recent large population based surveys have improved our understanding of the high prevalence and significant consequences of mental disorders. [1,2,5-8] These studies have identified a range of socio-demographic factors, behaviors, and traits that may be associated with the presence of common disorders including life stressors, female gender, past or family history[9], and personality traits (in particular neuroticism, self esteem and self criticism). [10,11] Some of these surveys have also attempted to establish a picture of the life course of these disorders by inquiring about the lifetime history of symptoms. [12]

However, retrospective approaches are limited in their ability to portray lifetime patterns of anxiety and depressive disorders since they are dependent on participant recall of past symptoms. This may be limited, particularly for nonpsychotic disorders. [13] Longitudinal, research is better placed to characterize the course of these disorders and to investigate the determinants of incidence, recurrence and prognosis. An increasing number of prospective studies are being undertaken to explore these issues, although the methods applied to this research are in their infancy and there has been little debate on the approaches that are widely used. The goal of this paper is to examine the methods commonly taken by longitudinal, population-based, psychiatric epidemiologic studies, to explore the conceptual and methodological issues arising from these studies, and to discuss the implications of these issues for future research designs.

## Methods

Much longitudinal research in this field has relied on brief "screening" measures focused on a single affective disorder such as depression. It would be impossible for any one paper to adequately describe the diversity of approaches taken in this body of research, let alone critique the study methods. A more limited number of population based studies have used comprehensive diagnostic measures which can identify a range of mental disorders. These more complex instruments allow examination of comorbidity and movement from one diagnostic category to another. Since a better understanding of these issues is vital for future research, and since the methodological issues that arise from research using complex instruments are relevant to all longitudinal studies in mental health, we undertook a review of longitudinal studies of mental disorders using structured diagnostic instruments. We use examples from this pool of research to highlight methodological and conceptual challenges that confront all researchers using longitudinal approaches in this field.

### Search strategy

We searched MEDLINE for articles including the key words "depression" or "anxiety disorders" in the following key word subheadings: diagnosis, epidemiology, ethnology, etiology, genetics and prevention & control. We also included all articles with the words "depression", "anxiety disorders" or "mental disorders" in the title or abstract. We limited our search to studies on humans and published in English language journals between 1990 and January 2007. 119,939 articles were identified. We further limited our review to the 14610 studies that either included the words "longitudinal", "cohort" or "prospective" in the abstract or title, or included "longitudinal studies", "cohort studies" or "prospective studies" as key words. We then restricted these to studies using structured diagnostic instruments and thus including either "Diagnostic Interview Schedule", "Composite International Diagnostic Interview" or "Clinical Interview Schedule" in the title or abstract. A total of 287 publications were found meeting these criteria. We identified all studies in adults (but including studies starting in earlier life) that had an initial cohort of over 1000 subjects drawn from the general population at first interview, with a minimum of one further interview. Studies needed to use a structured diagnostic instrument for the whole sample in at least two waves, and samples drawn from primary care or outpatients clinics were excluded. A summary of the key studies identified is shown in Table 1. We have not attempted to summarize the wealth of findings this body of research has generated, except where it is relevant to our discussion of methods. We particularly focus on incidence estimates, as this allows us to later explore key methodological issues. Where a study resulted in multiple outputs, we have only cited those most relevant to the point being raised.

**Table 1:** Key longitudinal studies discussed

**Study**	**Country**	**Years**	**Instrument**	**Follow-up ** **rate**	**Waves**	**Baseline Age ** **Range**	**Initial cohort ** **size**
Dunedin Multidisciplinary Health and Development Study (DMHDS) (Krueger et al., 1998)	New Zealand	1972 -	Modified DIS	92.7%	10	Birth cohort	1037
Christchurch Health and Development Study (Fergusson and Horwood, 2001)	New Zealand	1977 -	Modified DIS for Children; CIDI component	80%	21	Birth cohort	1265
Epidemiologic Catchment Study (ECA) (Eaton et al., 1984)	USA	1981–1982	DIS	78–83%	2	18 years and older	10,861 10,185
Baltimore ECA (Chen et al., 2000)	USA	1981 – 1994	DIS	73%	3	18 years and older (small oversampling of elderly)	3481
New Haven ECA	USA	1980–1982	DIS or CESD	81%	2	18 years and older (large oversampling of elderly)	5034 (1977 elderly)
National Comorbidity Study – 2 (Kessler, Merikangas et al., 2003b)	US	1990–2002	CIDI	76.6%	2	15–54 years	8098
Michigan Community Sample (Breslau et al., 2005)	US	1989–2001	DIS	89%	4	21–30 years	1007
Early Developmental Stages of Psychopathology (EDSP) (Stein et al., 2001)	Germany	1994–2004	Munich CIDI	84%	2 (plus 10 year follow-up of partial wave)	14–24 years	3021
Northern Rivers Mental Health Study (NoRMHS) (Beard et al., 2006)	Australia	1996–1998	CIDI	69%	2 (plus screening)	18 years and older	1407
Netherlands Mental Health Survey and Incidence Study (NEMESIS) (De Graaf et al., 2002)	Nether-lands	1996–1999	CIDI	65%	3	18–64 years	7076
British National Household Survey (Brugha et al., 2005) (Skapinakis et al., 2006)	UK	2000–2002	CIS-R	68%	3	16–74 years	3536 (stratified sample of first wave)
Aldona prospective study (Patel et al., 2006)	India	2001–2003	CIS-R	87%	3	18–50 years	2494 women

## Results

The United States Epidemiological Catchment Area (ECA) Program remains a benchmark in this field. The study involved five sites, with a total sample size of over 15,000 participants 18 years or older. Participants were first interviewed in 1981, followed by a second interview after 6 months at one site, and a 6 month telephone interview followed by a repeat mental health interview at one year for other sites. [14,15] Follow-up rates ranged from 78 to 83%. The Baltimore arm of the ECA study undertook further reinterviews between 1993 and 1996, with 73% follow-up of survivors.

The initial follow-up found 12 month first incidence (i.e. excluding participants with previous history) of disorders ranging from 1.59 per 100 person years for depression to 3.98 per 100 person years for phobic disorders. [16] However, when participants were followed up for longer periods in the Baltimore ECA, incidence rates dropped markedly with the annual incidence of first depression falling to 3.0 per 1000 person years. Severe depression was associated with female gender and family history, but not stressful life events, while mild and moderate depression was associated with family history and stressful life events, but not female gender. [17,18]

More recently, the Netherlands Mental Health Survey and Incidence Study (NEMESIS) followed randomly selected participants aged from 18–64 years from the Dutch adult population with assessments in 1996, 1997 and 1999. [18] 7076 participants were recruited to the first wave (69.7% of eligible persons), with 4796 followed over all three waves. In multivariate analysis, NEMESIS found that a high neuroticism score, female gender and negative life events or ongoing difficulties increased the chance of a participant developing depression, as did sleep problems at baseline. For anxiety, female gender, negative life events or ongoing difficulties and baseline symptoms or presence of a mood disorder were all significant predictors. For participants with depression at baseline, predictors of persistence included severity and low social support. [19] NEMESIS estimated the first incidence of DSM-III-R disorders at 5.6 (95 percent confidence interval:4.8–6.4) per 100 person years, with an incidence for depression of 3.1 per 100 person years (95% CI: 2.6–3.6). [20]

In Germany, the Early Developmental Stages of Psychopathology Study recruited 3021 participants 14–24 years of age in 1995 (response rate 71%). A sample of younger participants was initially reassessed, and then reinterviews were attempted with all participants 42 months after baseline (2548 participants reinterviewed – follow-up rate 84%). A 10 year follow-up has recently been completed. [21] The investigators used the Munich CIDI to explore outcomes including suicidality, the course of post traumatic stress disorder, the influence of cannabis use on psychotic symptoms, the role of trauma and PTSD on premenstrual disorders, and the predictive significance of anxiety disorders on first onset of major depression. [22-26]

In the US, the National Co-morbidity Study (NCS) of 8098 participants aged 15–54 years reinterviewed 4375 participants (follow-up rate 76.6%) approximately 10 years after their initial interview and papers from this cohort are just starting to become available. [27] In the UK, a stratified sample of the original 8000 respondents to the 2000 Psychiatric Morbidity Survey (all of those with a definite or sub-threshold psychiatric disorder and a 20% random sample of those without such disorder) were contacted eighteen months later for a follow-up interview. 2413 of 3536 were successfully re-interviewed (68% response rate). [28] This cohort has been used to explore a range of possible social determinants of mental health.

Other longitudinal studies have focused on specific population subsections including a cohort of 1200 21 to 30 year old members of a large health maintenance organization in southeast Michigan followed over 4 waves (899 participants interviewed in all waves)[29], and a three wave study of 2494 women aged 18–50 years in India. [30]

One weakness of these large studies is that they have difficulty accurately determining either prior history, the sequence in which these disorders may develop, or the influence on their development of a range of childhood experiences. To do this, detailed information going back to early childhood is required. Two cohorts in New Zealand have been followed over extended periods from birth to early adult life, allowing investigation of these issues.

The Christchurch Health and Development Study (CHDS) is a longitudinal study of a birth cohort of 1265 children born in the Christchurch urban region during mid 1977. [31] Participants have been studied at repeated (until age 16, annual) intervals across their life course, including detailed assessment of mental health outcomes using the DIS and, later, the CIDI. Loss to follow-up over this period has been limited to approximately 20%. This study has been able to investigate a number of crucial issues in mental health. For example, it has suggested that the influence of childhood experiences such as divorce or separation on later mental health is less important than the influence of events leading up to separation, that a predominantly homosexual sexual orientation may be associated with greater risk of depression[32], and that the risk of suicidal behavior depends on accumulative exposure to adverse childhood circumstances, personality factors, mental disorders and exposure to adverse life events.

The Dunedin Multidisciplinary Health and Development Study is a similar birth cohort study conducted in New Zealand, following 1037 participants with regular assessments to age 26, and using the Diagnostic Interview Schedule for assessment of mental health status. [33] Loss to follow-up at age 21 was limited to an extraordinary 7.3%. Among many other findings, this study suggested a close link between personality traits and the later development of mental disorders [34] and, more recently, identified an interaction of the serotonin transporter (5-HTT) gene on the influence of life stressors on incident depression. [35]

While studies using brief instruments are too numerous to detail in this paper, their findings are generally consistent with those of the studies outlined above that have used more comprehensive structured interviews, although their estimates of incidence and prevalence have tended to be higher. [36] They include an extensive research program in twins that has proposed comprehensive developmental models for depression that explain approximately half of the variance in the liability to depressive episodes. [37,38]

### Key questions that could be answered by longitudinal studies

While this extensive body of research is fundamental to our current understanding of mental health, fundamental questions remain (Table 2).

**Table 2:** Some important questions that can be answered by longitudinal research

**Characterization of affective disorders**	
Symptoms across the life course	What is the lifetime prevalence of disorders?
	Do disorders tend to appear in a particular order?
	Is there a prodrome?
	Are there distinct trajectory subtypes and do these have the same aetiology?
	Are there distinct subtypes of symptom patterns over time?
Classification of disorder	Are current classifications adequate or are there higher order structures that better represent affective disorders and their evolution over time?
**Aetiology of affective disorders**	
Determinants of recurrence	Are the factors that influence disorder recurrence or transformation to new disorder/comorbidity the same as those that determine incident symptoms?
Influence of environmental factors	Does an individual's physical and social environment influence changes in symptom levels or type?
Genetic research	Do specific syndrome or trajectory subtypes have distinct genetic determinants?
Heterogeneity	Do specific syndrome or trajectory subtypes have differing etiology?

### Disorders across the life course

We still have only a limited understanding of the natural course of these disorders across a lifetime. The birth cohort studies have shed considerable light on the evolution of mental disorders in childhood adolescence and early adulthood, but these are small cohorts and patterns beyond early adulthood are less clear. The Baltimore ECA suggests a secondary peak in incidence of depression around the mid 50's. [39] If this could be confirmed, it would be useful to understand why this occurs and whether it can be prevented. It is also unclear what happens to the prevalence of mental disorders in older age. Studies using brief instruments suggest higher rates of depression, but this may be due to higher sensitivity of the measures used. [40] The limited longitudinal research that has followed participants into older age suggests a slight increase in prevalence, but this may simply be a response to increasing disability. [41,42]

### Classification of disorders

There has been longstanding debate on whether current diagnostic categories of affective disorders accurately reflect the complexity of these conditions. Many individuals suffer comorbidity from more than one mental disorder and many individuals with a "pure" disorder without comorbidity later develop other disorders. These patterns are a challenge for longitudinal research, but prospective studies also provide an ideal vehicle to further our understanding of these issues and to help resolve debate as to the nosology and etiology of these conditions. [43]

At least two perspectives have been suggested, with some researchers tending to favor the existing categorical approach, and others looking to higher order structures in a search for common etiologic and pathologic processes. Factor analysis of the National Comorbidity Survey has been used to promote the latter approach, suggesting an alternative model in which disorders are divided into those representing externalizing or internalizing problems with the latter having two sub factors – anxious-misery and fear. [44] These findings are supported by two prospective studies. [45,46] However, a large prospective study of adolescents found that anxiety disorders generally preceded the onset of depression, and that the risk factors for both were distinct. [47] A recent analysis of the Christchurch cohort examined changing symptoms over three waves and found that both perspectives may be valid: participants appeared to have symptoms correlated across the study period that were consistent with broad generalized internalizing, but there was also evidence of across-time continuity in disorder-specific components of symptoms. [48]

Longitudinal research can help our understanding of the nature of common mental disorders and our system of classification. However, to do this, information is needed on the full range of affective symptoms in participants at relatively frequent intervals over long follow up periods. This is currently limited by the lack of dimensional symptom measures for all participants in current structured instruments (see below) and the inability of studies using a single brief measure to capture comorbidity patterns.

### Determinants of recurrence

Much of the focus of recent longitudinal research has been to identify the incidence and determinants of new diagnoses of mental disorders in participants without a prior history. However, such analysis has generally depended on participant recall of past symptoms. There is little evidence to confirm the accuracy of recall of lifetime symptoms, test retest studies suggest it may be surprisingly high for psychotic symptoms, but less so for nonpsychotic disorders. [13] This concern is supported by the Baltimore ECA, which found 17 of the 22 episodes of depression reported at the 1982 interview were not reported at the 14 year follow-up. [39]

The bias that is likely to result from these recall limitations is clearly demonstrated in the estimates of the onset ("incidence") of new disorders reported by different studies. Initial first incidence estimates of depression in the Baltimore ECA dropped markedly when longer follow-up allowed more objective assessment of psychiatric history. This presumably reflects participants who may have self reported no prior history but who were excluded on more objective assessment. The incidence of depression found by NEMESIS was similar to the original ECA results, reflecting reliance on self reported history, while the incidence in studies of participants with and without past history, is double this. [49]

It is important to better characterize the true incidence of first onset disorders and identify the factors associated with them. However, the Baltimore ECA findings suggest that first onset of depression after early adulthood is relatively rare. From a public health perspective, the burden of disease associated with first onset of disorders after early adulthood is far less than that posed by recurrence of these disorders (or worsening of symptoms) throughout the rest of life. Yet little research has explored the factors that influence disorder recurrence. These may, or may not, be distinct from those that influence first incidence.

In practical terms, excluding participants with prior history from analyses results in a considerable loss of information and power. From a public health standpoint, there is a clear imperative to identify modifiable factors that impact on the recurrence of disorder or exacerbation of symptoms in the high proportion of the population with a lifetime history.

### Heterogeneity of disorders

Clinical experience suggests that affective disorders are heterogeneous in nature. A neglected area of longitudinal research is whether different symptom patterns may be associated with different etiologic pathways and prognoses. A number of studies have used latent class analysis to identify possible depression sub classes. One study of twins identified three clinically significant depressive syndromes: mild typical depression, atypical depression, and severe typical depression. [50] Individuals with recurrent episodes tended to have the same syndrome on each occasion and genetic makeup appeared to be a significant factor in this tendency. Latent Class Analysis of the National Comorbidity Survey found four slightly different clinical subtypes for depression: mild/severe and typical/atypical. [51] Importantly, the association of various risk factors differed by subtype, with atypicality being associated with interpersonal dependency and reduced self esteem. The Baltimore ECA also used Latent Class Analysis to identify depressive subtypes. [17] Severity was associated with female gender and family history but not stressful life events, while mild or moderate cases were associated with family history and stressful events, but not female gender.

These findings are significant since they suggest that differing subtypes of specific affective disorders (for example individuals with symptoms over time of both depression and generalized anxiety disorder) may vary in their susceptibility to particular risk factors. If these disorders are categorized as homogeneous entities, these relationships may be obscured in analysis. Longitudinal analysis with the power to follow different subgroups over time may be able to identify risk factors for recurrence that are specific for disorder type.

### Disorder trajectories

Longitudinal research also offers the potential to examine changing patterns (trajectories) of symptoms over time. [41,52-55] The limited research in this field has generally been consistent with a trait-state model where depression symptomatology is accounted for by two factors: an underlying "trait" effect that is highly heritable and reflects underlying vulnerability, and a residual "state" effect that is less inheritable and more likely to reflect circumstances at a point in time. Distinguishing these patterns is another area that is worthy of future research since it may be more appropriate for etiologic studies to examine the influence of external factors on "state" effects rather than the more stable "trait" effects. It is also possible that some disorder subclasses (for example individuals who over time may experience both depressive and generalized anxiety disorders) may experience different vulnerabilities to particular risk factors than others (for example an individual with no such comorbidity). Distinguishing these comorbidities over time in analysis may give us a better understanding of the risk factors for these individuals.

### Influence of environmental factors

Our understanding of how the physical and social environment influences the recurrence of mental disorders is also very limited. A number of neighborhood level factors, including social disorder, neighborhood safety, social connectedness and neighborhood socioeconomic status or disparity have been suggested as possible influences on the risk of an individual developing an affective disorder. [56-58] This might be because they potentially serve a role ameliorating stressful exposures, or may themselves comprise one. Longitudinal studies are particularly important in this area, since associations identified in cross-sectional research may simply reflect a tendency for people with recurring disorders to congregate in particular areas, or have similar social habits, rather than these associations being causal in nature. While the large cohort studies have considerably increased our understanding of the individual level determinants of common mental disorders, few have explored in depth the influence of neighborhood level factors. Where factors from the physical and social environment have been considered by these studies and those using brief instruments, these have generally relied on participant self report which may itself be influenced by confounding factors such as age, gender, symptom severity or personality traits. Large studies with more objective measures of these complex factors are needed to help us better understand the role of these potentially modifiable environmental determinants.

### Genetic research

It is beyond this paper to examine in detail the potential arising from recent advances in our understanding of the human genome. However, association studies for mental disorders have tended to be based on cross sectional disorder categorizations and have generally considered affective disorders as homogeneous entities. [59] Yet, symptom and trajectory subtypes are likely to reflect quite distinct genetic susceptibilities. [50] Stratifying association studies by these subtypes may be a productive avenue for future research. Using longitudinal studies to identify subclasses of affective disorders is therefore likely to also advance our search for their genetic determinants.

Similarly, by allowing populations to be subtyped by genetic susceptibility, advances in genetic research may offer great potential for longitudinal research into individual and environmental risk factors. [60] Gene-environment interaction approaches that better characterize genetic influences on individual susceptibility and explore the heterogeneity in response to environmental precipitants are likely to be particularly fruitful. [59]

### Moving Forward

A number of design elements need to be considered if longitudinal studies are to answer these, and other, important questions. As a starting point, more studies need to be conducted in large samples of the general population, with symptoms of both anxiety and depression assessed repeatedly over an extended period.

### Measurement of outcomes

While some researchers have expressed concern that the validity of structured diagnostic instruments is stronger for high prevalence clinical settings[61] than in non-clinical populations [62,63], there is considerable evidence that positive diagnoses in population surveys using these instruments correlate well with disability [64,65].

However, in longitudinal research it is also important that *changes *in diagnosis identified by these instruments reflect meaningful changes in symptoms rather than minor symptom changes in participants lying just above or below diagnostic cutoffs. For example, an individual with several symptoms of depression but not meeting one of the key criteria might simply change this one response to shift from a negative to a positive diagnosis. It is conceivable that respondents who experience several waves of interviews may learn to answer no to such gateway questions as a way of shortening the interview. There has been little assessment of the strength or validity of instruments such as the World Mental Health(WMH)-CIDI to measure change. Some reassurance comes from Australian research in which over three quarters of participants developing a new diagnosis had one or no symptoms at baseline, while three quarters of those meeting all symptom criteria at baseline were depression free two years later [49]. While this may partly reflect the stem structure of the CIDI instrument, such major shifts in symptom levels seem likely to reflect clinically meaningful changes. However, there is very limited additional evidence to support this conclusion and this is an area worthy of further examination.

A related issue is the reliance of these instruments on categorical outcomes (i.e. diagnoses of depression or anxiety disorders). While this is useful for examining the natural course of mental disorders, it may be less than ideal when exploring their determinants. For example, the Christchurch study, found "evidence of continuous and generally linear dose-response functions between symptom severity and outcome risks and that dimensionally scored variables were considerably better predictors of outcome than measures based on diagnostic classification". [66] The NEMESIS study also found subthreshold expressions of depression and (hypo)mania to be continuous with more clinical states and that they strongly predicted post baseline mood disorders. [67] Limiting analysis to considering mental disorders as categorical outcomes is thus wasteful of information and weakens study power. [68]

To address this need, the WMH-CIDI now incorporates a dimensional measure of depressive symptoms, the Quick Inventory of Depressive Symptomatology – Self Report (QIDS-SR). [69] However, this is only invoked if a stem depression question is positive limiting the ability to examine symptom change in all responders over time. Another limitation of structured diagnostic instruments is their length, which in some cases may take several hours, with a high associated cost of administration. This can influence both sample size and frequency of follow-up.

Brief measures have the potential to address many of these problems in longitudinal research, and some have been surprisingly well validated. For example, in validation against structured mental health professional interviews, the Patient Health Questionnaire (PHQ), a 9 item depression measure, had a sensitivity of 88% for major depression with a specificity of 88%. [70] A more recent validation of the PHQ against the Structured Clinical Interview for DSM-IV (SCID) found 90% sensitivity and 83% specificity for diagnosis of major depressive disorder. [71] This compares favorably with the assessment of non psychiatrically trained physicians (sensitivity 40%; specificity, 87%) and with the CIDI (overall concordance with SCID of 87%). [72]

However, an issue that is of particular concern with brief instruments arises from the potential for comorbidity of mental disorders. A brief instrument for a specific outcome such as depression is not capable of distinguishing individuals who may also be suffering from a separate, possibly related, disorder such as generalized anxiety disorder. This prevents examination of the relation between these outcomes over time and also limits genuine assessment of new onsets. For example, individuals identified by a brief instrument as being depression free may, in fact, have unidentified symptoms of an anxiety disorder and a high level of psychological distress. It is debatable whether it is reasonable to consider symptoms of depression that develop in such an individual as being a true new onset of psychopathology.

One solution may be multi-outcome brief measures which could be used to categorize participants for both depression and anxiety disorders. The PHQ, for example, has an additional anxiety component. However, the complexity of anxiety disorders means these brief instruments can generally only assess symptoms of some anxiety disorders, leaving participants with other disorders (e.g. obsessive compulsive disorder) unidentified. With this approach, the limitations of categorical analysis also continue.

As with structured instruments, there are considerable advantages for brief instruments that can measure changes in symptom levels rather than just the presence or absence of diagnoses. A number of brief dimensional depression measures, including the PHQ and the QIDS-SR, have been shown to be sensitive to symptom change. Anxiety measures, such as the State Trait Anxiety Inventory, that are less tied to specific DSM-IV diagnoses and capture both underlying anxiety trends and those more related to immediate circumstances, may be the best option for dimensional assessment of anxiety symptoms. [73] However, how symptom levels in both these domains can be aggregated in a single outcome is unclear.

An alternate approach may be measures of non-specific psychological distress, which are effective screening scales for "serious mental illness" and are both widely used and relatively robust. [74] These measures are dimensional and can be used to assess changes in symptoms levels over time, but do not provide specific information on diagnostic categories. They may, however, add value when combined with another measure. For example, by measuring psychological distress in addition to depression, it should be possible to identify participants who do not meet the criteria for depression, but who are likely to have another disorder such as anxiety.

### Measurement of covariates

Exposure to recent stressors has been repeatedly associated with new onsets of disorders and is usually included in each wave of a longitudinal study in the form of an assessment of major life events. [75] Personality trait neuroticism has also been consistently associated with increased risk and may reflect underlying susceptibility partly resulting from genetic makeup and childhood experience. [20,76] It can be assessed with a brief questionnaire. [77]

A range of other individual and environmental covariates have been included in recent studies including family history, the extent of social networks,[78] physical activity,[79] and history of childhood sexual abuse. [80]

Effects of the physical and social environment can be considered by asking study participants to report on characteristics of their homes, social networks and neighborhood. [58] More objective environmental information can be gathered directly by researchers, or from secondary data. [81] Past analyses have often considered the impact of neighborhood-level socioeconomic disadvantage (for example mean household income). While interesting, this is a broad measure that reflects more the characteristics of the neighborhood population than structural features that are amenable to intervention. Examining the impact of other characteristics such as crime levels, neighborhood disorder and urban design has been positive for outcomes such as walking[82-84]and may be worth considering as a contributor to mental health. However, neighborhood factors such as these, and those available from the Census, tend to be highly collinear and need to be considered in constructs that are consistent with theories of neighborhood effects. One way to do this is using factor analysis to derive a parsimonious and uncorrelated set of factors that capture the key neighborhood socio-economic and socio-demographic measures of interest. [85,86]

### Analytical approaches

Having dimensional information from multiple waves allows approaches to analysis that are not possible when examining diagnostic changes over a more limited follow-up. However, one issue that needs to be borne in mind when considering and communicating the complex findings that arise from repeated longitudinal data is total survey error. The potential for sampling error, coverage error, measurement error and non-response error to influence study findings is compounded in such research and any limitations arising from these need to be clearly communicated.

At its most simple, analyses of longitudinal datasets can examine the predictors of change in symptom scores for a particular disorder. Descriptive analyses could also explore how symptoms of both anxiety and depression vary over time.

Latent Class Analysis can be used to determine whether certain symptoms tend to cluster in subtypes within, or across, disorders[51], while latent growth models[87] and their extension; general growth mixture models [88] can be used to measure symptom trajectories. Longitudinal analysis could then stratify by these categories to examine whether the influence of covariates of interest varies by subtype.

Having data available for two covariates (for example symptoms of depression and physical activity) over several study waves, makes it possible to explore whether change in one of these variables, preceded change in the other. One way of doing this is with Dual Change Score Models, which distinguish true from error variance independently for the two variables of analysis, model the variables' systematic change, and simultaneously include competing hypotheses about lead-lag effects between the two variables. [89,90] They can also estimate the systematic change patterns of both variables, each variable's autoproportional effect, and the coupling effect that each variable may exert on the changes of the other variables. [91]

Where environmental exposures are identified at a neighborhood level, examination of environmental effects will often require multilevel models. [92,93] These can be used to estimate the fixed effects of neighborhood-level predictors as well as within- and between-neighborhood variability in individual-level outcomes. When considering neighborhood effects, the smaller the spatial unit, the greater the power. [94] However, it is also important to ensure that these areas have coherence with neighborhood boundaries that are meaningful to those who live within them.

## Conclusion

Many important, and in some respects quite basic, questions remain about the life course of depression and anxiety disorders and the determinants of their incidence, recurrence and prognosis. Much research to date has been based in a diagnostic paradigm that reflects clinical experience. A greater focus on symptoms and changes in symptom severity has the potential to allow innovative approaches that may greatly increase our understanding of these important issues. These include better characterising the heterogeneity of disorders by considering symptom subtypes and trajectories, and by examining the relationship over time between symptoms of different affective disorders. Considering these conditions as dimensional, heterogeneous, entities may then allow us to identify individual and environmental factors that impinge only on specific subtypes. It is also likely to advance our search for genetic determinants of these common disorders.

While we have focused in this paper on the extant literature from studies using comprehensive diagnostic instruments, these issues are just as relevant to research using brief instruments. Indeed, the cost and efficiency of such studies means they may be best placed to answer some of the questions confronting us. However, if we are to significantly further our understanding of these disorders, it is crucial that, regardless of the instruments used, future longitudinal research better addresses the issues of comorbidity and heterogeneity.

## Abbreviations

CHDS: Christchurch Health and Development Study; CIDI: Composite International Diagnostic Interview; DSM: Diagnostic and Statistical Manual of Mental Disorders; ECA: Epidemiological Catchment Area; NCS: National Co-morbidity Study; NEMESIS: Netherlands Mental Health Survey and Incidence Study; PHQ: Patient Health Questionnaire; PTSD: Posttraumatic Stress Disorder; QIDS-SR: Quick Inventory of Depressive Symptomatology – Self Report; SCID: Structured Clinical Interview for DSM; WMH-CIDI: World Mental Health

## Competing interests

The authors declare that they have no competing interests.

## Authors' contributions

JB conceived of the paper and oversaw manuscript preparation. SG helped develop the ideas discussed in the paper and contributed to manuscript preparation. DV contributed to debate and manuscript preparation.

## Pre-publication history

The pre-publication history for this paper can be accessed here:


